# Generation of red light with intense photoluminescence assisted by Forster resonance energy transfer from Znq_2_ and DCM thin films

**DOI:** 10.1007/s11356-022-23217-z

**Published:** 2022-10-03

**Authors:** Amina Laouid, Amine Alaoui Belghiti, Krzysztof Wisniewski, Abdelowahed Hajjaji, Bouchta Sahraoui, Anna Zawadzka

**Affiliations:** 1 Institute of Physics, Faculty of Physics, Astronomy and Informatics, Nicolaus Copernicus University in Toruń, Grudziadzka 5, PL 87-100 Torun, Poland; 2 National School of Applied Sciences, Engineering Science for Energy Laboratory, Chouaib Doukkali University of El Jadida, El Jadida, Morocco; 3 Centrer for Modern Interdisciplinary Technologies, Nicolaus Copernicus University in Toruń, Wilenska 4, PL 87-100 Torun, Poland; 4 LPHIA, SFR MATRIX, University of Angers, Physics Department, 2 Bd Lavoisier, 49045 ANGERS cedex 2, France

**Keywords:** Photoluminescence, Decay time, Thin film, DCM, Znq_2_, Physical vapor deposition, AFM

## Abstract

In this work, a novel experimental investigation of photoluminescence properties of Znq_2_ thin films co-doped with different concentrations of DCM were performed. The thin films were successfully deposited on glass substrates with different compositions, under high vacuum, by using the vacuum evaporation technique. For all compositions, the photoluminescence was measured at room temperature and also at low temperature in a wide range from 77 to 300 K with a step of 25 K in a high vacuum. The lifetime of the sample studied in real time was also measured using the decay time technique. The results obtained confirm that the doping influences the intensity of the DCM photoluminescence and also shows a complete energy transfer occurred from Znq_2_ to DCM which may have shifted the photoluminescence peak from Znq_2_ to the orange wavelength region which is related to DCM. The lifetime of the sample studied in real time was about 4.47 ns for Znq_2_ and while all the other samples showed two decay time components. As a result, the doping influences the optical properties of Znq_2_ and makes it a potential candidate for optoelectronic applications.

**Graphical abstract:**

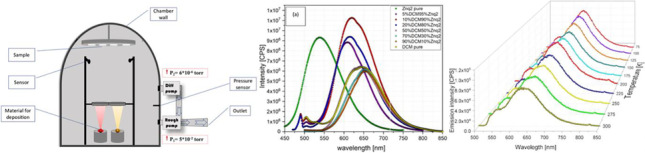

## Introduction

Organic materials have become among the major research topics lately (Data and Takeda 2019; Yu et al. 2021); its use has given rise to promising applications at the industrial scale and opened up new perspectives for fundamental physics (engineering at the molecular scale), because of their broad potential benefits in various fields such as optoelectronics (Kukhta and Bryce 2021), environmental purification (Xie et al. 2020; Wang et al. 2019), biomaterials (Zhou et al. 2020), solar energy conversion (Lee et al. 2017), sensors (Pięk et al. 2018), and in the field of medicine (Alam et al. 2018). These materials can combine very interesting optical (absorption, emission), electronic (insulating, semiconductor, metallic, or superconducting), and mechanical properties in the form of a single molecule, self-assembling single layers, or thin crystalline or amorphous films. The manufacture of thin-film has attracted considerable attention in the world in recent years due to its uses in different applications for different fields of research (Pan et al. 2019; Saadiah et al. 2019).

Organic dyes, in particular merocyanines dyes (Schembri et al. 2021), (Kulinich et al. 2018), and the metal–organic materials in the form of thin films, have been widely used in the last decades in various applications as energy transport materials, in particular for optoelectronic devices, dye lasers, organic light-emitting diode (OLED), and solar cells, because of their interesting electrical and optical properties (Liess et al. 2019; Li et al. 2021; Pozin et al. 2021; Saeed et al. 2020; Gu and Zhang 2019; Ou et al. 2019).

4-(Dicyanomethylene)-2-methyl-6-(4-dimethylaminostyryl)-4H-pyran (DCM) is an organic coloring molecule that belongs to the class of merocyanines; it was first used by Eastman Kodak Company in 1997 as a doping material in the red color laser (Hong et al. 2021). This molecule contains a push–pull system which generally consists of an electron donor part and an electron acceptor part that are separated by a conjugated bridge, and this structure has attracted the attention of several research groups. It has shown great potential in its field due to its very interesting photophysical properties like its high fluorescence quantum efficiency, its absorption, and emission spectra, and also it has shown a large Stokes shift (Laouid et al. 2022). Therefore, it received great interest, which made it a basic material for several applications such as luminescent solar concentrators, photovoltaic, inorganic light-emitting diodes, and NLO applications (Popczyk et al. 2019; Waszkowska et al. 2020). This molecule contains a push–pull system which generally consists of a donor and an acceptor part of electrons (Noirbent et al. 2021). It has shown great potential in its field because of its very interesting photophysical properties (Weishäupl et al. 2021).

Studies on metal–organic materials, particularly metal–organic materials based on 8-hydroxyquinoline, remain a subject of interest due to their interesting and unique properties known for their fluorescence strength and significant non-linear optical properties (Kutluay 2021; Chen et al. 2022; About et al. 2021). Among these hydroxyquinoline compounds, we have a green luminescent material called bis(8-hydroxyquinoline) zinc (Znq_2_). It is a semiconductor material that has attracted great interest from several researches’ teams because of its mechanical, electrical, thermal, and chemical properties (Saito et al. 2021; Shinde et al. 2018; Li et al. 2019; Lougdali et al. 2022). Znq_2_ has shown in front of aluminum tris (8-hydroxyquinoline) (Alq_3_), which is the leader in its field in terms of luminescence efficiency and high quantum efficiency at a low operating voltage (Shahedi et al. 2017). This makes it an attractive material in various applications, such as medicine, photovoltaics, photonics, optoelectronics, and especially for OLED applications (Yuan et al. 2021; Shahedi et al. 2021).

On the other part, photoluminescence is a field that has been expanding and refining since the 1970s (Gilliland 1997). Photoluminescence spectroscopy is a powerful non-destructive optical technique, which allows to characterize semiconductor materials and also insulating materials (Yu and McCluskey 2021). Because of its good resolution, high precision, and high sensitivity, it has become an important technique in the study and engineering of materials. Time-resolved photoluminescence (TRPL) is a technique adapted to study the quick electronic deactivation processes that lead to the emission of photons in many types of materials such as metal–organic complexes and dyes. This fluorescence lifetime can be influenced by several parameters such as the molecular environment as well as by interactions with other molecules. This technique makes it possible to measure life in real time.

The main objective of this work is to manufacture organic thin films that are both environmentally friendly and represent a suitable candidate for optoelectronic applications; for this purpose, thin films of the different compositions of DCM and Znq_2_ were developed using the vacuum evaporation technique in order to take advantage of the properties presented by these two materials. This paper is centered on the study of the impact of doping and temperature on photoluminescence and decay time, and also studied the transitions made to its various samples. The photoluminescence results obtained showed that the mixture of these organic compounds (DCM and Znq_2_) present a very good alternative to produce optoelectronic devices and more particularly a very good one to generate red color lasers of good quality and very low cost.

## Experimental methods

### Deposition technique of thin films

Since the glass surface quality has an impact on the properties of the thin films, cleaning is of considerable importance before starting the deposition. The cleaning method used consists of using an acetone bath with ultrasound for 15 min, then an ethanol bath, and finally, cleaning with isopropanol using flow synthesis.

The different samples were deposited on glass substrates using the vacuum evaporation technique under high vacuum 5 × 10^−6^; the high vacuum is created by using two-pump diffusion pump and roughing pump is using the following system System-NANO 36™ (Kurt J. Lesker Company) (Popczyk et al. 2019).

**Fig. 1:**
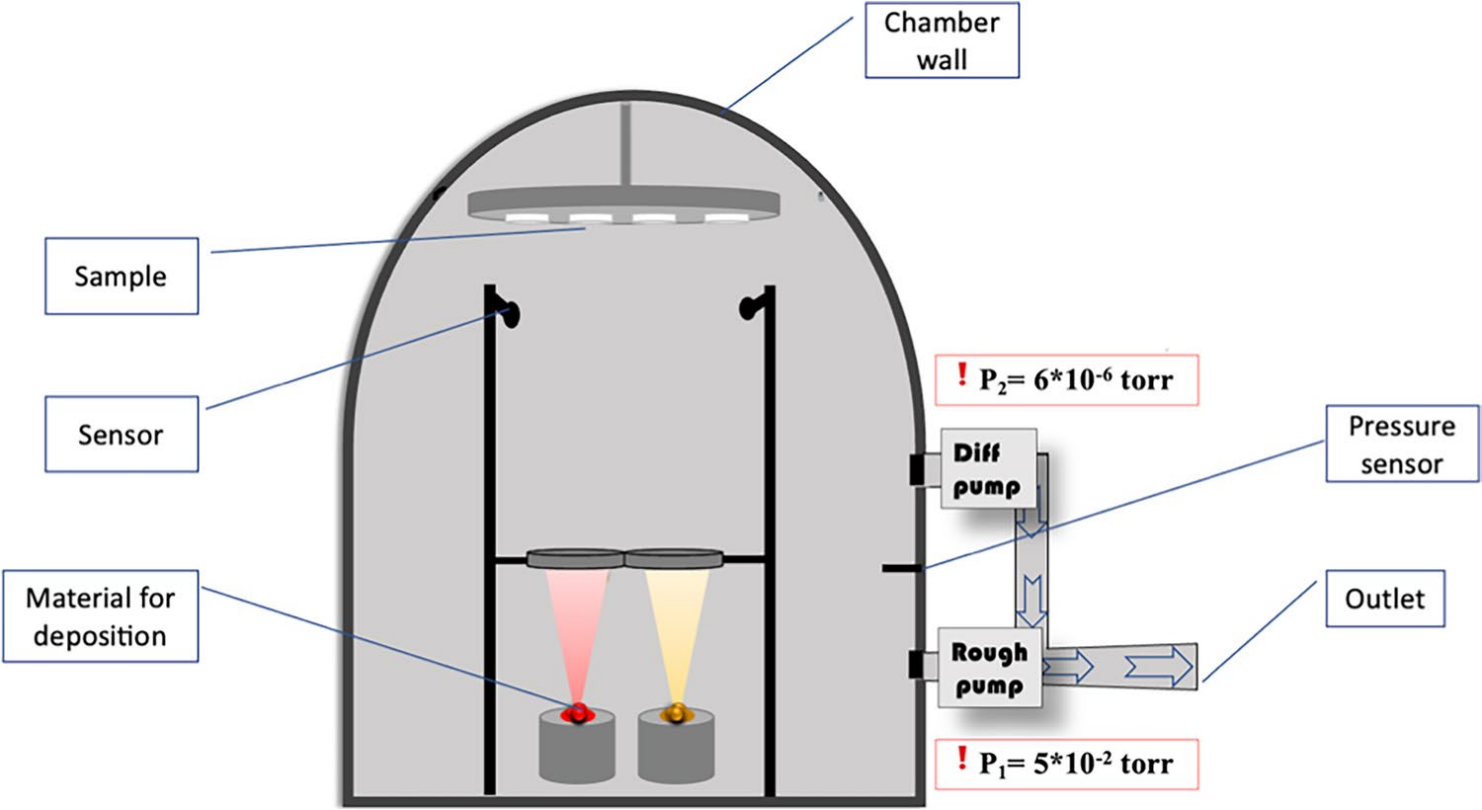
Physical vapor deposition (PVD) apparatus

Figure 1 shows the diagram of the co-deposition vacuum evaporation process. Powdered materials were purchased from Sigma-Aldrich and used without any purification. In order to obtain the optimal conditions for the deposition carried out in this experiment, the powders were placed separately in two aluminum oxide crucibles. Each crucible is placed in turn in a shielded tungsten crucible heater to control the temperature of the materials so that they have different deposition temperatures. The temperatures were controlled manually. The substrates are placed in a rotating holder. This study aims to deposit samples with different compositions and therefore the adjustment of the rates is a very important element; for this reason, the crucibles were covered until the rate control to ensure that the desired percentages were deposited, after adjusting all the parameters including density, Z-factor, and thickness of each element; checking the machine parameters, and also reaching the secondary vacuum which is another necessary parameter for the deposition as it provides a guarantee of film purity. The deposition process starts, and the powder starts to evaporate to form the thin film on the substrates. After obtaining the desired thickness (100 nm) with the help of the sensors, the deposition process stops automatically.

### Characterization of thin films

In the present work, the atomic force microscopy technique (AFM) was used to examine the morphological properties of DCM co-doped Znq_2_ thin films deposited on glass substrates. AFM imaging was studied in tapping mode, using the fiber-lite MI-150 equipment.

The photoluminescence of the thin layers grown on the glass substrate was measured by using FluoroMax-4 spectrofluorometer using FluorEssence software, and the source of excitation was a xenon lamp (Anoua et al. 2021; Zawadzka et al. 2019). This machine allows the measurement of excitation and emission as a function of the wavelength.

**Fig. 2:**
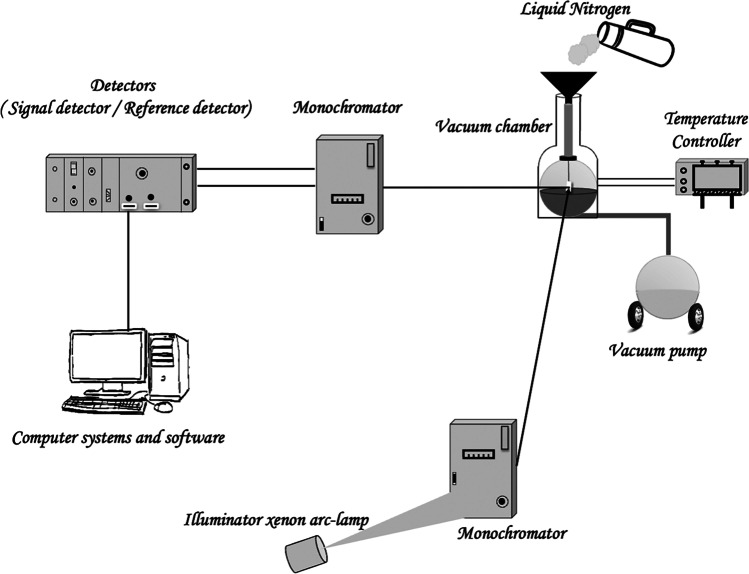
Experimental setup for low-temperature photoluminescence measurements

In order to study carefully the influence of the low temperature on the photoluminescence intensity of the samples, the measurements were carried out in the temperature interval of 77 to 300 K with a step of 25 K. The cooling was performed using liquid nitrogen. The photoluminescence measurements were made inside a vacuum chamber under vacuum 10^−3^ torr as it is presented in Fig. 2. The substrate was attached to a temperature controller to adjust the temperature at each time.

## Results and discussion

### Morphological proprieties

The AFM pictures, roughness, and profile of each composition are presented in Fig. 3, Fig. 4, Fig. 5, Fig. 6, Fig. 7, Fig. 8, Fig. 9, and Fig. 10.

**Fig. 3:**
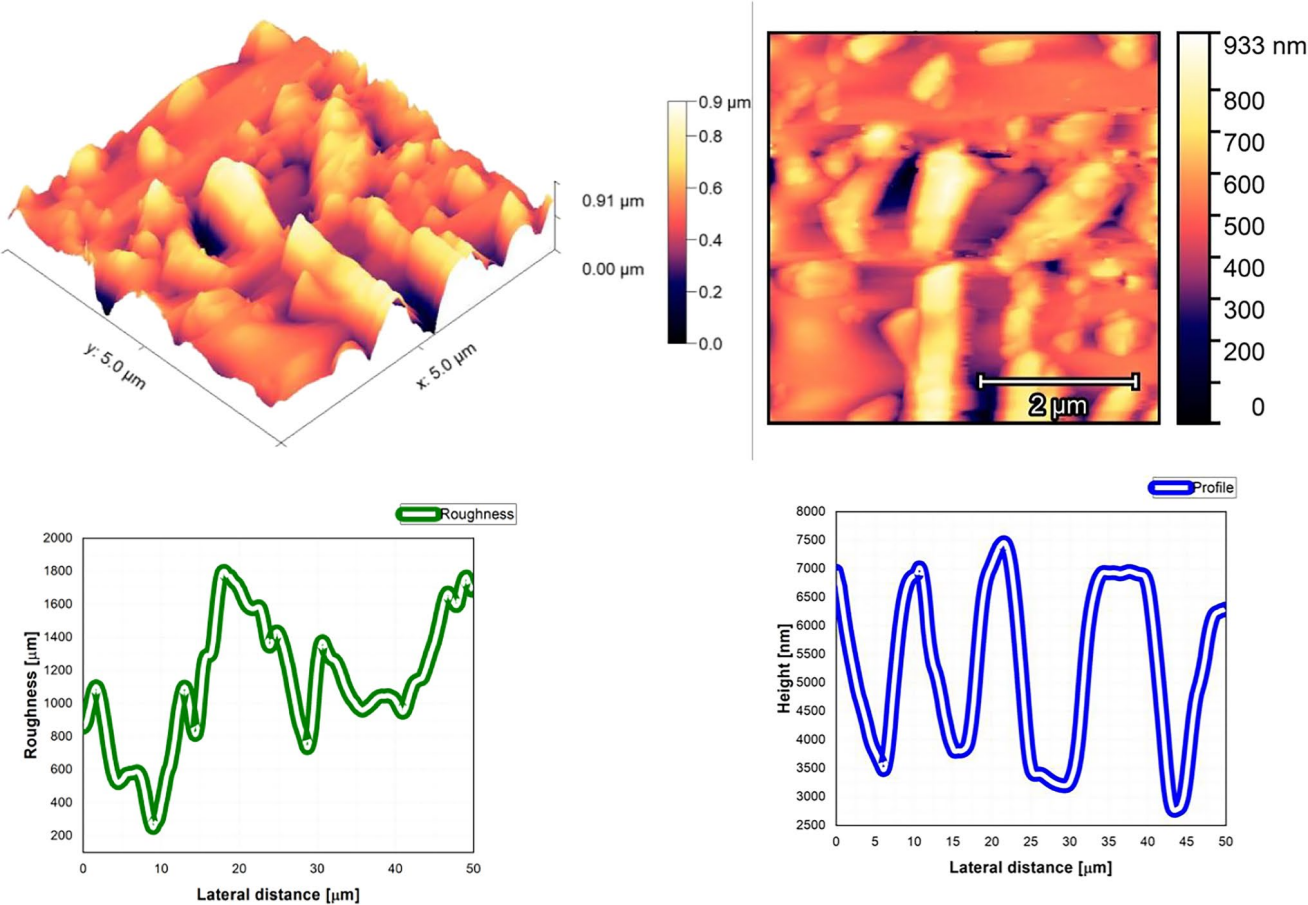
AFM image, roughness and profile of DCM pure thin film (5 µm × 5 µm)

**Fig. 4:**
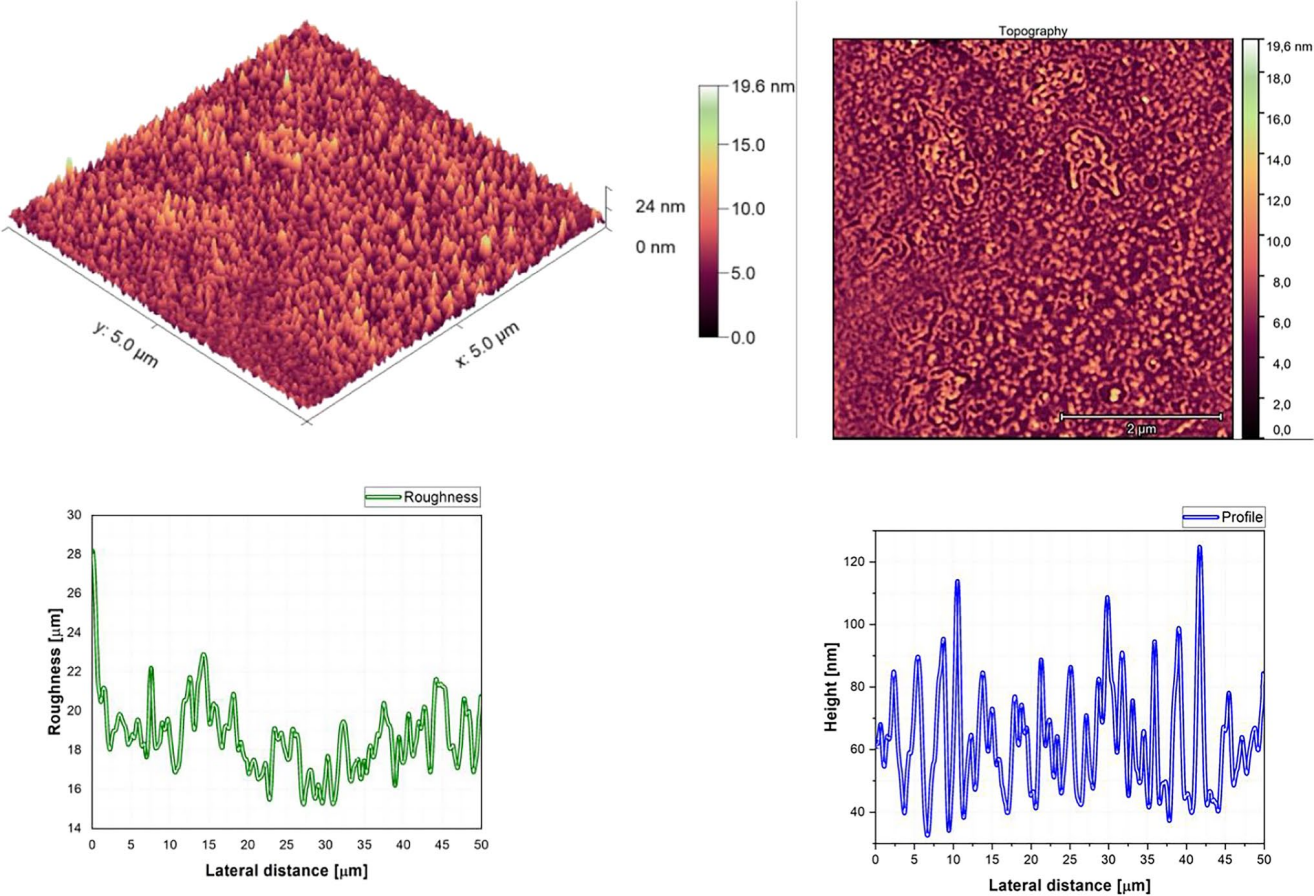
AFM image, roughness and profile of Znq_2_ pure thin film (5 µm × 5 µm)

**Fig. 5:**
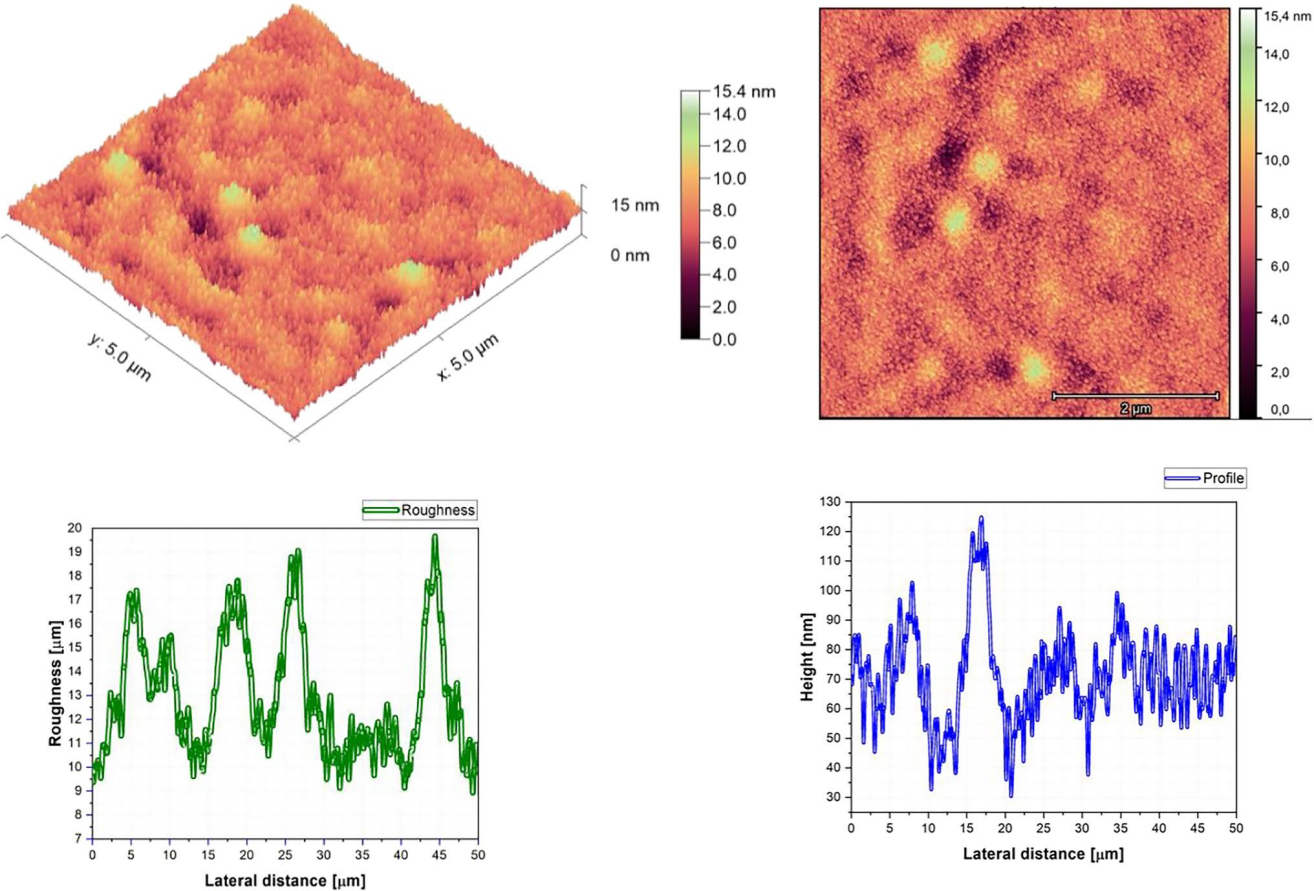
AFM image, roughness and profile of 90%DCM-10%Znq_2_ thin film (5 µm × 5 µm)

**Fig. 6:**
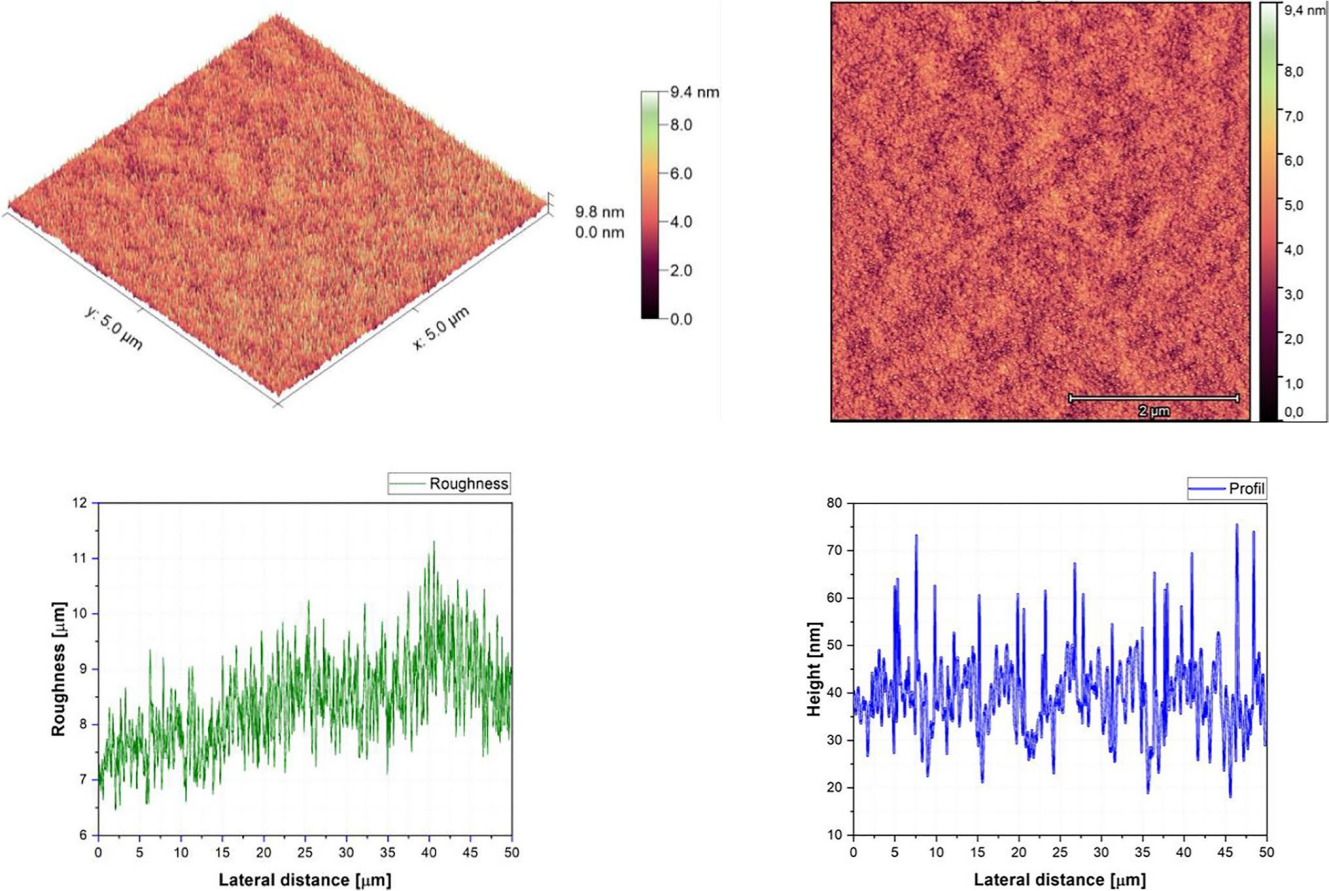
AFM image, roughness and profile of 70%DCM-30%Znq_2_ thin film (5 µm × 5 µm)

**Fig. 7:**
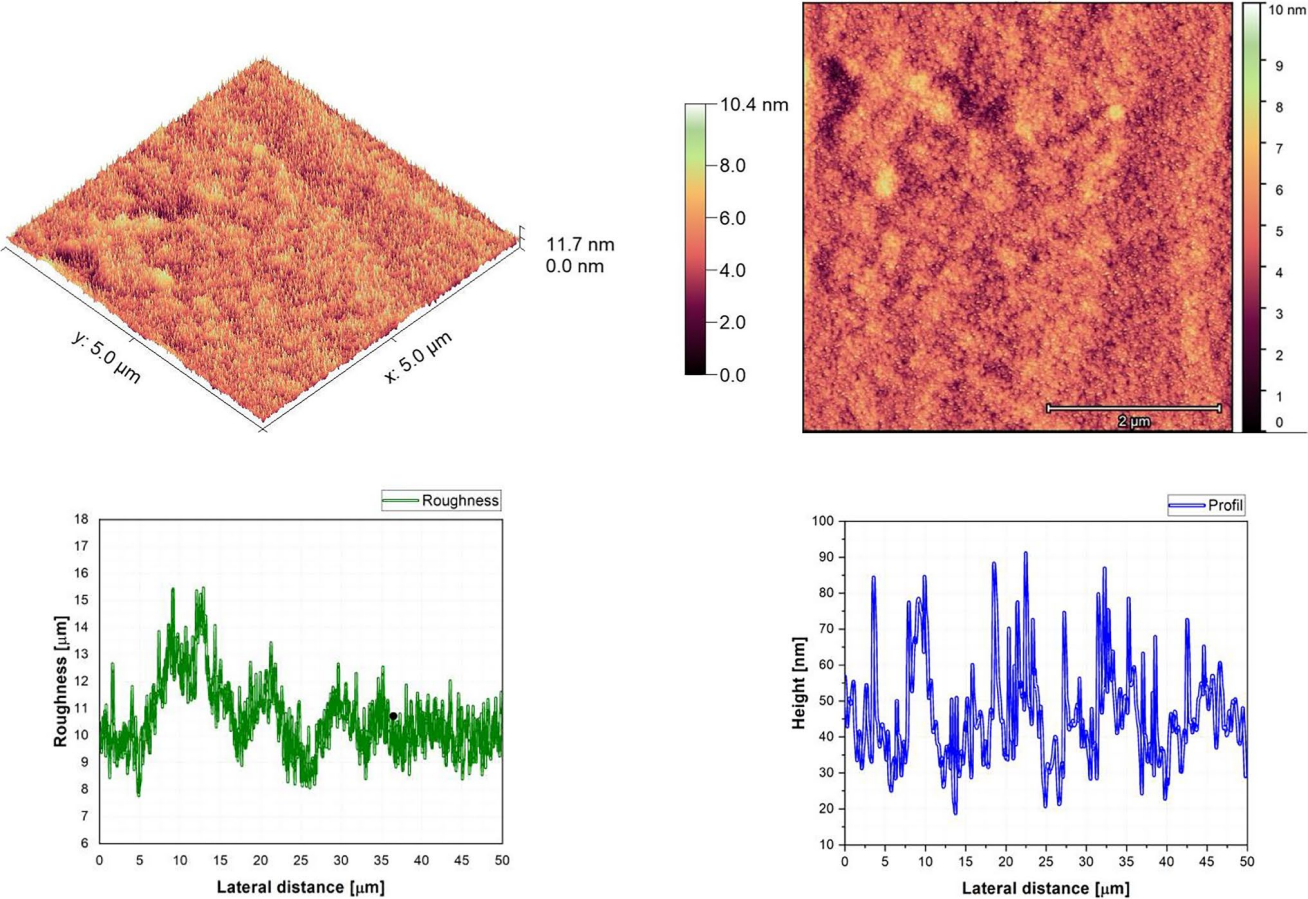
AFM image, roughness and profile of 50%DCM-50%Znq_2_ thin film (5 µm × 5 µm)

**Fig. 8:**
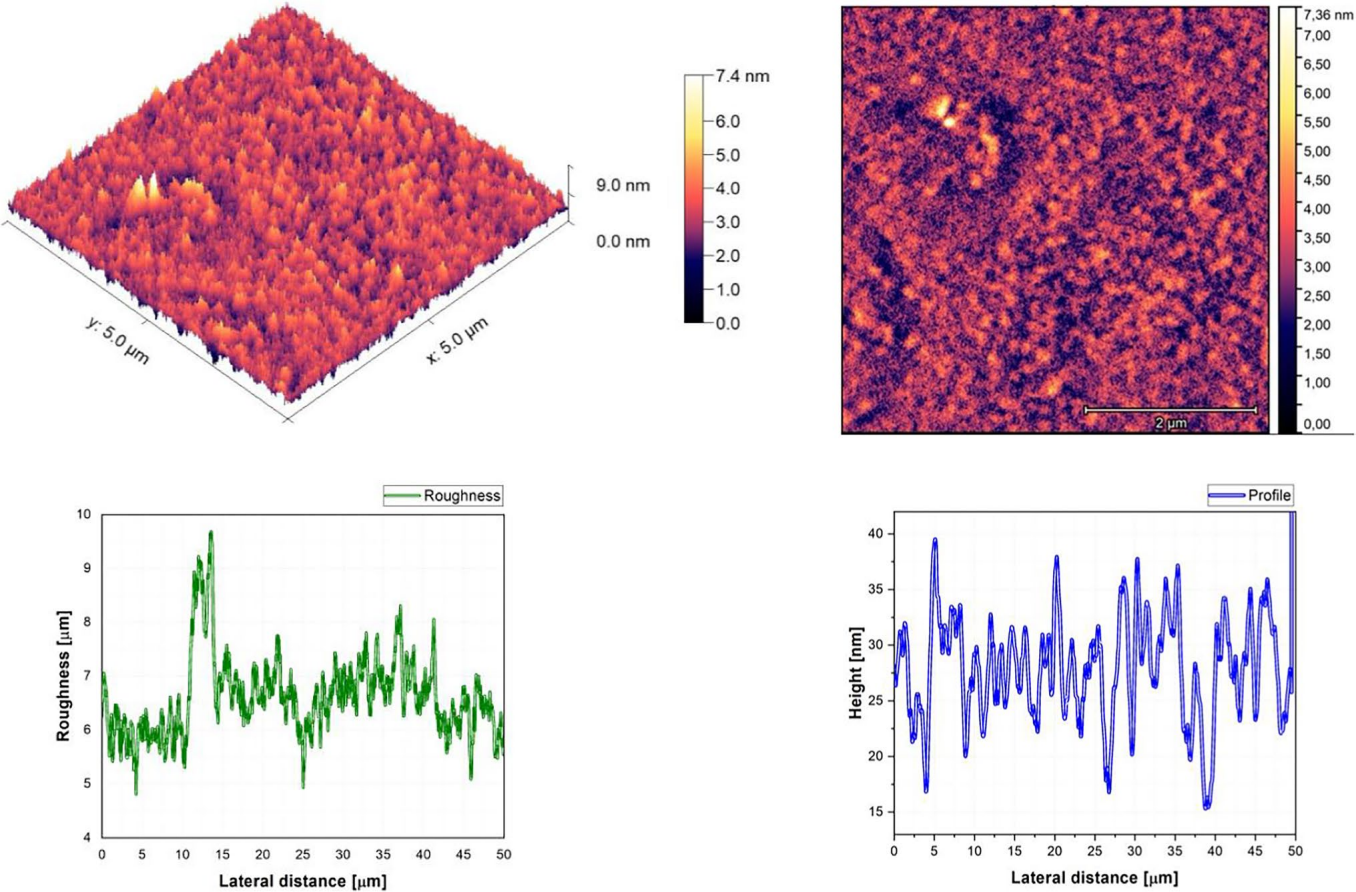
AFM image, roughness and profile of 20%DCM-80%Znq_2_ thin film (5 µm × 5 µm)

**Fig. 9:**
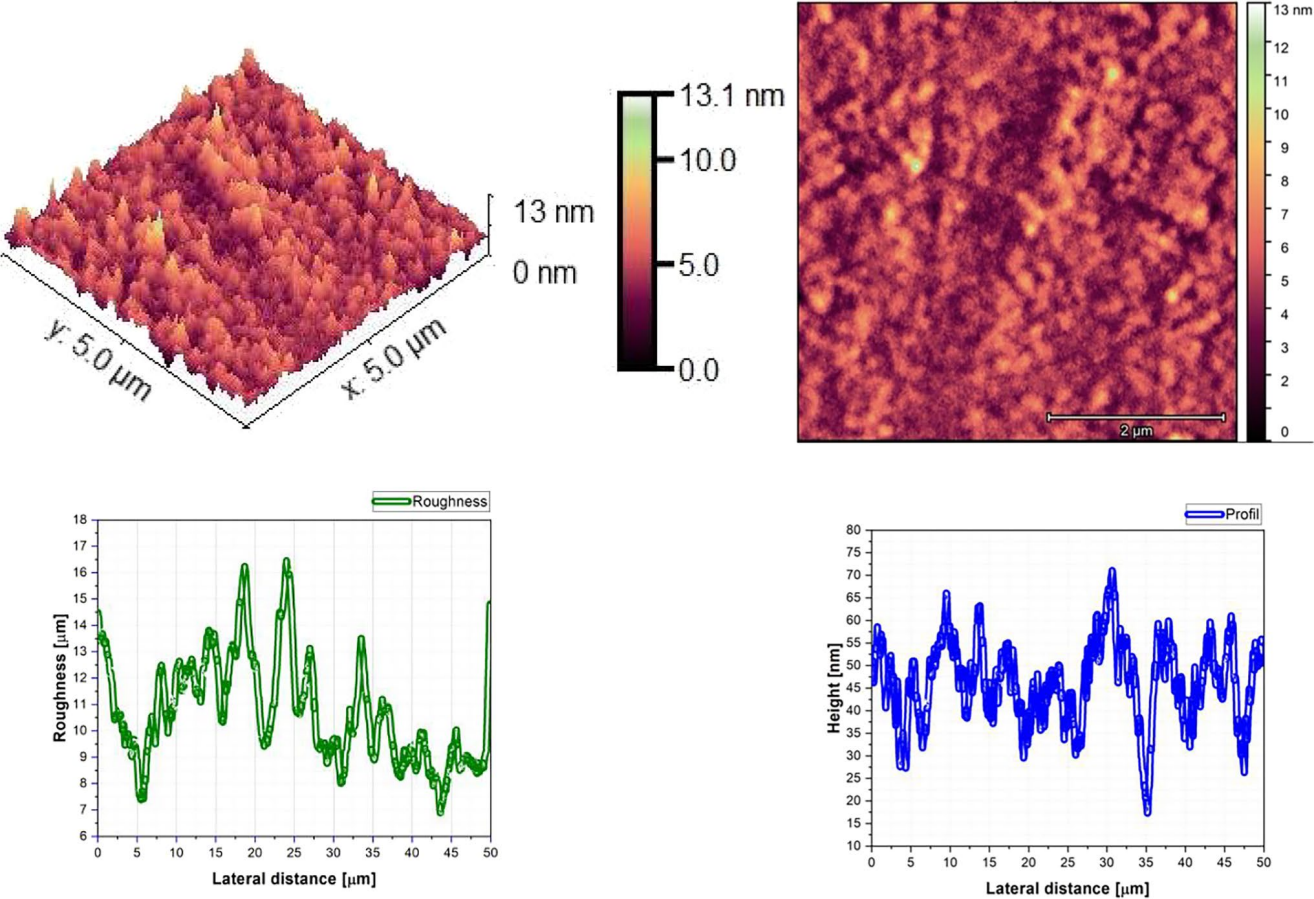
AFM image, roughness and profile of 10%DCM-90%Znq_2_ thin film (5 µm × 5 µm)

**Fig. 10:**
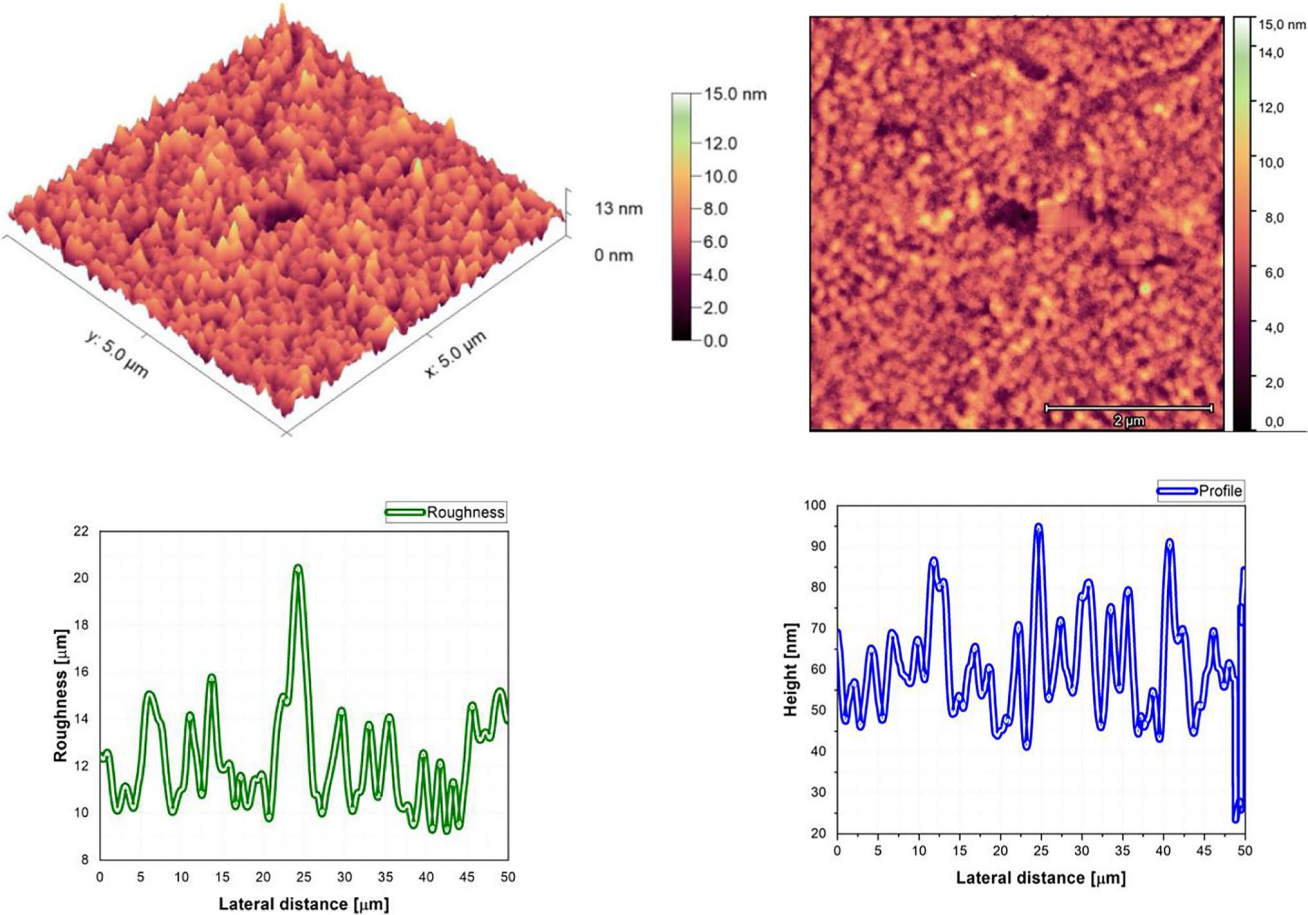
AFM image, roughness and profile of 5%DCM- 95%Znq_2_ thin film (5 µm × 5 µm)

The AFM pictures demonstrate that doping influences the surface of the samples. For the images of the pure DCM sample, they showed a highly roughened (Average value $\approx 1$.12 $\mathrm{\mu m}$) surface with microscopic grains as shown in the film profile, while the sample of the pure Znq_2_ showed a sponge structure with low thickness and manometric size granules are particularly noticeable. The other samples showed homogeneous morphology with a tendency to form nano spherical grains of a few nanometers in height as shown in the film’s profiles. The samples of 90%DCM-10%Znq_2_, 70%DCM-30%Znq_2_, and 50%DCM-50%Znq_2_ showed a flat surface with average roughness values respectively 12.9 nm, 9 nm, and 10 nm, while the samples of 20%DCM-80%Znq_2_, 10%DCM-90%Znq_2_, and 5%DCM-95%Znq_2_ showed a sponge surface structure as in the case of pure Znq_2_ with average roughness values respectively 7 nm, 10 nm, and 12 nm.

### Photoluminescence at room temperature

For all samples except Znq_2_, the emission spectrum measurements were studied from 500 to 850 nm, excited by 450 nm, while the excitation spectrum was measured in the range 300 to 600 nm at the maximum value of the emission intensity of about 645 nm. For thin films of Znq_2_, its emission spectrum was studied from 450 to 700 nm, excited by 395 nm, while the excitation spectrum was measured in the range 200 to 500 nm at the maximum value of the emission intensity of about 536 nm.

**Fig. 11:**
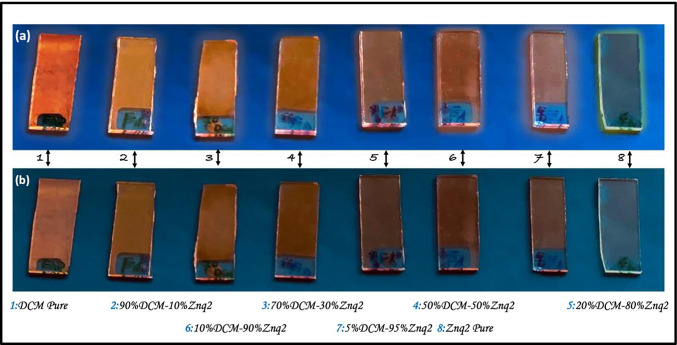
The actual fluorescence changes of the Znq_2_ Co-doped DCM samples. (**a**) The samples exposed to ultraviolet light and (**b**) the samples under normal conditions

The fluorescence changes of Znq_2_ samples co-doped with DCM are shown in real images in Fig. 11.

**Fig. 12:**
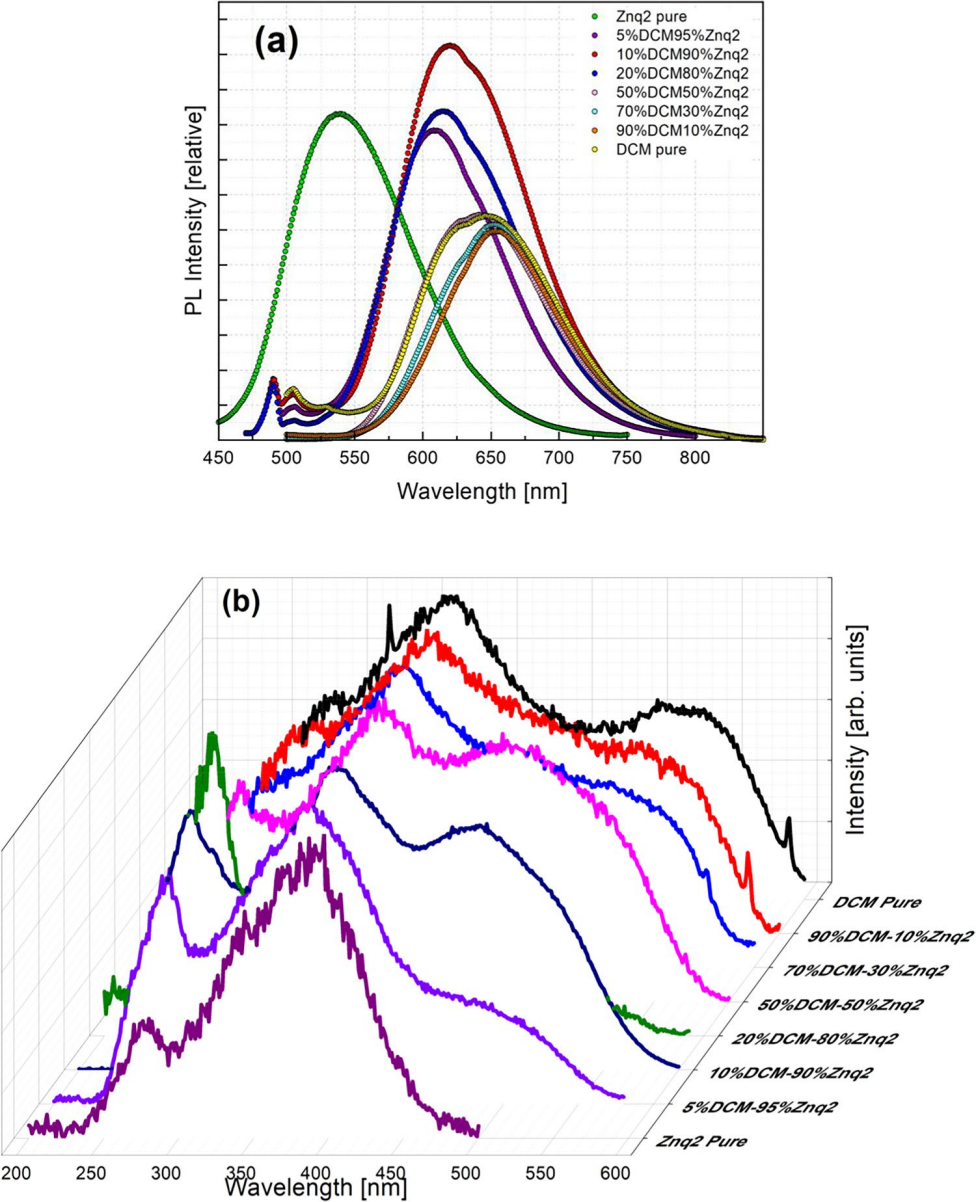
**a** Emission, **b** excitation spectra at room temperature for all the samples

The photoluminescence spectra of Znq_2_-doped DCM thin films with different percentages are shown in Fig. 12.

The pure Znq_2_ thin film showed strong luminescence in advance of the pure DCM thin film, while the highest luminescence intensity is that of the 5%DCM-95%Znq_2_ sample. For the other samples, the intensity of photoluminescence is diminished compared to the pure Znq_2_ thin film as the percentage of Znq_2_ increases in the structure. For samples with a large percentage of Znq_2_ (5%DCM-95%Znq_2_, 10%DCM-20%Znq_2_, 20%DCM-80%Znq_2_), their PL spectra were shifted to orange (41 nm $\pm$ 5 nm) relative to the pure Znq_2_ PL spectrum, and present three emission bands, as in the case of pure DCM. The first two bands had a low intensity at 490 nm and 504 nm respectively; however, the third most intense band is 614 nm. While in the samples with a large percentage of DCM (70%DCM-30%Znq_2_, 90%DCM-10%Znq_2_) their spectra were shifted to red (with 116 nm), 50%DCM-50%Znq_2_ sample has taken the character of pure DCM.

**Fig. 13:**
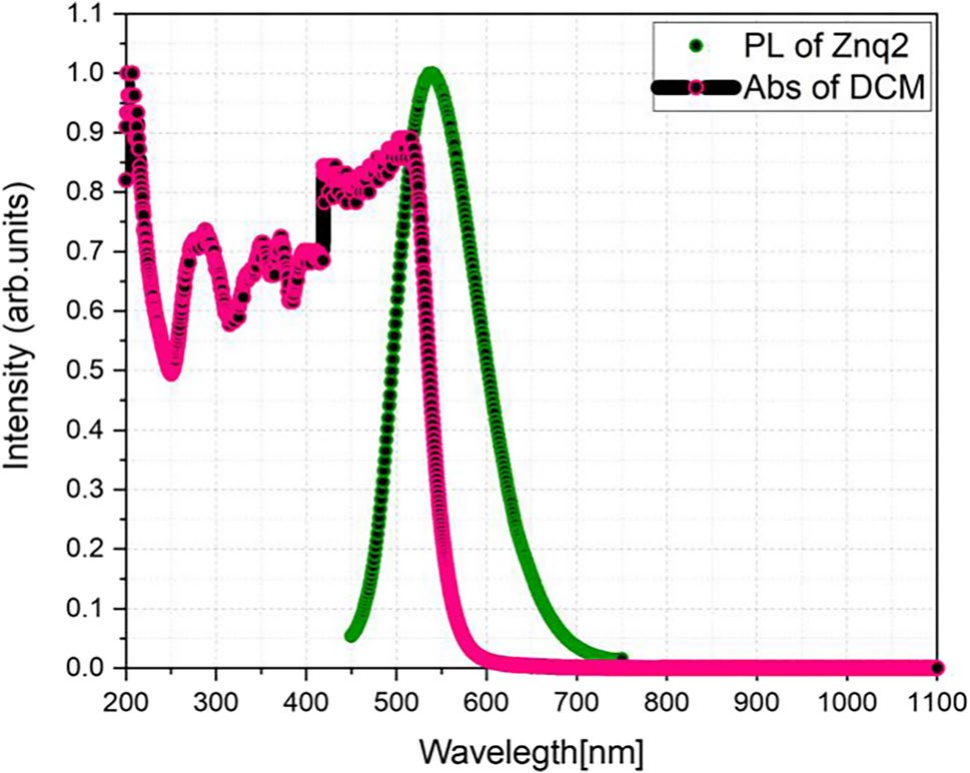
Photoluminescence characteristic of Znq_2_ and absorbance DCM

The samples with the different compositions of DCM and Znq_2_ show peaks in the orange color emission region which is related to DCM; this result indicates that a complete energy transfer has occurred from Znq_2_ to DCM due to the overlap between PL Znq_2_ emission and DCM absorption Fig. 13. This is the same result found by Abedi et al. (2014) when they combined DCM with Alq_3_.

**Fig. 14:**
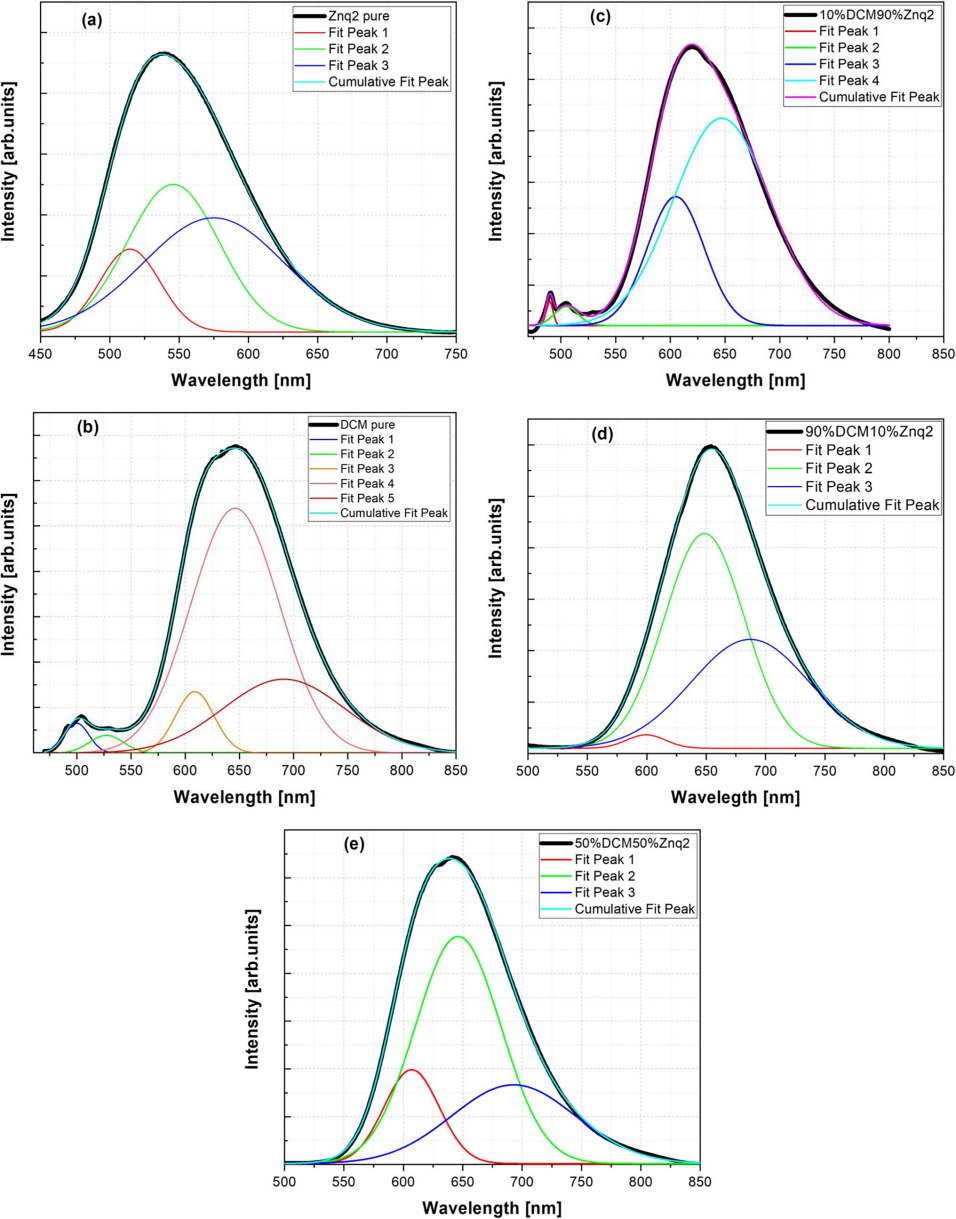
Curves fitting for emissions spectra at room temperature of **a** Znq_2_ pure, **b** DCM pure, **c** 10%DCM90%Znq_2_, **d** 90%DCM10%Znq_2_, **e** 50%DCM50%Znq_2_

As the observed PL spectra consist of a large band of visible luminescence, each spectrum was fitted with the Gaussian function in order to carefully study the transitions that occurred in the samples. The deconvoluted spectra involve several emission peaks, and each emission peak corresponds to a specific transition present in the particular sample; furthermore, the global spectrum is the sum of the corresponding Gaussian peaks. Figure 14 shows the deconvoluted PL spectra of all samples.

Through analysis of the fitted spectra, the pure DCM sample has presented five distinct characteristic peaks at or around 503 nm, 527 nm, 608 nm, 640 nm, and 692 nm. The pure Znq_2_ thin film shows three distinct characteristic peaks at or around 515 nm, 546 nm, and 579 nm. Samples with a large percentage of DCM showed three emission bands around 601 $\pm$ 4 nm, 648 nm, and 695 nm $\pm$ 5 nm, while samples with a large percentage of Znq_2_ have 4 emission bands around 490 nm, 506 $\pm$ 1 nm, 600 $\pm$ 4 nm, and 646 nm. Table 1 summarizes the results of the Gaussian fitting for emission spectra for all the samples at room temperature.

**Table 1:** Results of the Gaussian fitting for emission spectra for all the samples at room temperature. Xc in nm, height in CPS, and FWHM in nm

	Fit peak 1			Fit peak 2			Fit peak 3			Fit peak 4					Fit peak 5		
	Xc	Height	FWHM	Xc	Height	FWHM	Xc	Height	FWHM		Xc	Height	FWHM	Xc		Height	FWHM
DCM PURE	499	659535.14046	26.58845	527.33726	390638.97906	36.74496	608.82174	1,355355.86481	41.84463		640.03807	5396564.22015	96.8016	691.05649		1630946.77058	131.55236
Znq2	514.42161	2726313.05767	51.33698	545.69495	4854684.81121	78.70279	574.93722	3753124.97596	119.84472		–	–	–	–		–	–
5%DCM 95%Znq2	489.24916	1284663.27	7.98128	503.39739	769666.101	25.31	597.76339	4448217.32	63.59839		632.86541	5730050.93	105.46354	–		–	–
10%DCM 90%Znq2	489.6478	1386813.81	7.46383	504.92097	1224957.56	25.76946	604.85187	5429541.6	61.17927		646.85488	8508488.96	104.33478	–		–	–
20%DCM 80%Znq2	489.18938	1497170.35	9.08331	504.158687	603848.331	26.21	600.77929	5216751.56	66.99464		644.94822	6303056.03	106.33999	–		–	–
50%DCM 50%Znq2	–	–	–	–	–	–	606.92666	1990662.14	54.64879		646.07893	4787492.93	84.72181	692.82098		1679683.08	122.71296
70%DCM 30%Znq2	–	–	–	–	–	–	603.72772	582342.954	34.80091		648.48619	4892473.12	73.01478	695.92903		1800509.34	104.3869
90%DCM 10%Znq2	–	–	–	–	–	–	599.34227	261047.13556	38.55556		648.47274	4171336.11192	79.6351	687.07545		2114133.66569	117.65101

The emission bands of the pure DCM sample peaking at 503 nm, 527 nm, and 646 nm represent the characteristic bands, it can be attributed to the transition from HOMO → LUMO, HOMO-1 → LUMO, and HOMO → LUMO + 1. The other emission bands can be attributed to the excited and defect levels created in the film during deposition. The other thin films showed a dominance of red emission bands that are characteristic of pure DCM. They also showed new emission bands that depend on the percentage of Znq_2_ in the structure.

**Fig. 15:**
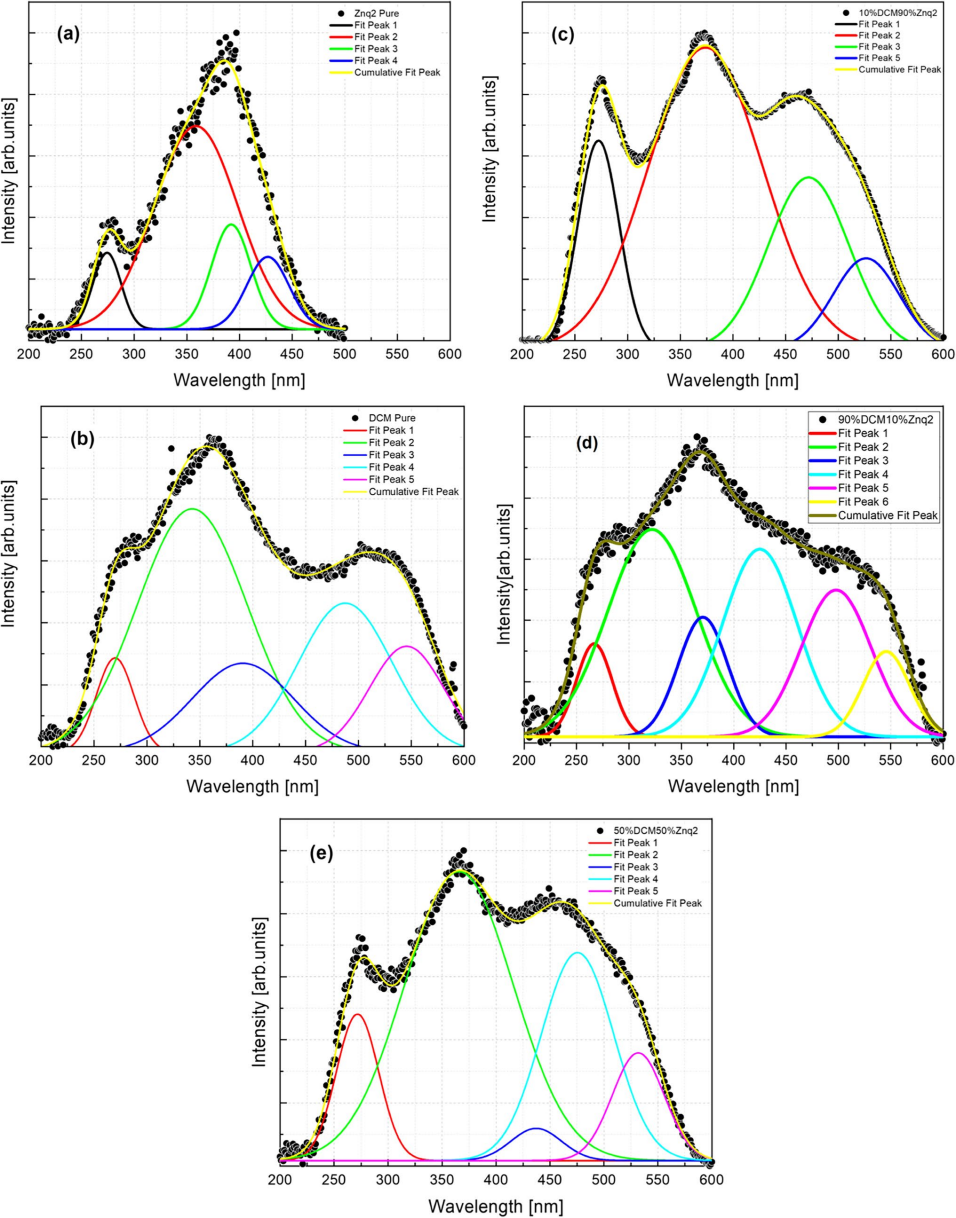
Curves fitting for excitations spectra at room temperature of **a** Znq_2_ pure, **b** DCM pure, **c** 10%DCM90%Znq_2_, **d** 90%DCM10%Znq_2_, **e** 50%DCM50%Znq_2_

The excitation spectra were also fitted by the Gaussian function, as shown in Fig. 15. The fitting of the excitations graphs allows to identify the transitions carried out inside the thin film; more precisely, it gives a piece of information on the energy of gape of the thin films. Table 2 below summarizes the peaks’ values found and their equivalent energies for all samples.

**Table 2:** Results of the Gaussian fitting for excitations spectra for all the samples at room temperature: peak in nm, Eg in eV

	Peak 1	E	Peak 2	E	Peak 3	E	Peak 4	E	Peak 5	E	Peak 6	E
*DCM pure*	269	4.6	319	3.8	366	3.38	408	3.03	509	2.43	550	2.25
*Znq2 pure*	270	4.59	356	3.5	392	3.16	427	2.9	–		–	
*90%DCM10%Znq*_*2*_	269	4.6	319	3.8	371	3.34	420	2.95	490	2.53	545	2.27
*70%DCM30%Znq*_*2*_	269	4.6	316	3.5	369	3.36	405	3.06	487	2.54	548	2.26
*50%DCM50%Znq*_*2*_	269	4.6	330	3.9	369	3.36	418	2.96	476	2.6	535	2.31
*20%DCM80%Znq*_*2*_	270	4.59	322	3.8	369	3.36	409	3.03	456	2.7	525	2.36
*10%DCM90%Znq*_*2*_	269	4.6	315	3.9	370	3.36	430	2.88	470	2.63	525	2.36
*5%DCM95%Znq*_*2*_	270	4.59	318	3.8	369	3.35	409	3.03	465	2.66	525	2.36

The pure Znq_2_ film showed the presence of 4 peaks, while pure DCM film showed six peaks. Pure Znq_2_ film has shown the transition that indicates their gape energy 2.9 eV (Monzon et al. 2011); this result is found by several research teams. The same for the pure DCM film has presented the transition of its gape energy 2.2 eV (Fujii et al. 1997; Qin et al. 2016).

The presence of the peak at 270 nm in all samples is well noted. The samples with different compositions, they presented from their turn six peaks. These peaks are also distinguished by the presence of the characteristic peaks of pure DCM and pure Znq_2_ and also distinguished by the presence of new peaks which are the characteristic peaks of these samples. These results allowed us to confirm the presence of several transitions in the thin films. The fact that DCM belongs to the push–pull system which is both electron donor and acceptor, and Znq_2_ has the character of an electron donor, the deposited films likely have a donor1-acceptor-donor2 chain form. And consequently, the structure has characteristic energy of pure DCM, of pure Znq_2_, and more energies that are most likely related to the transition due to the interaction between A-D1 and the interaction between A-D2.

### Temperature effect

**Fig. 16:**
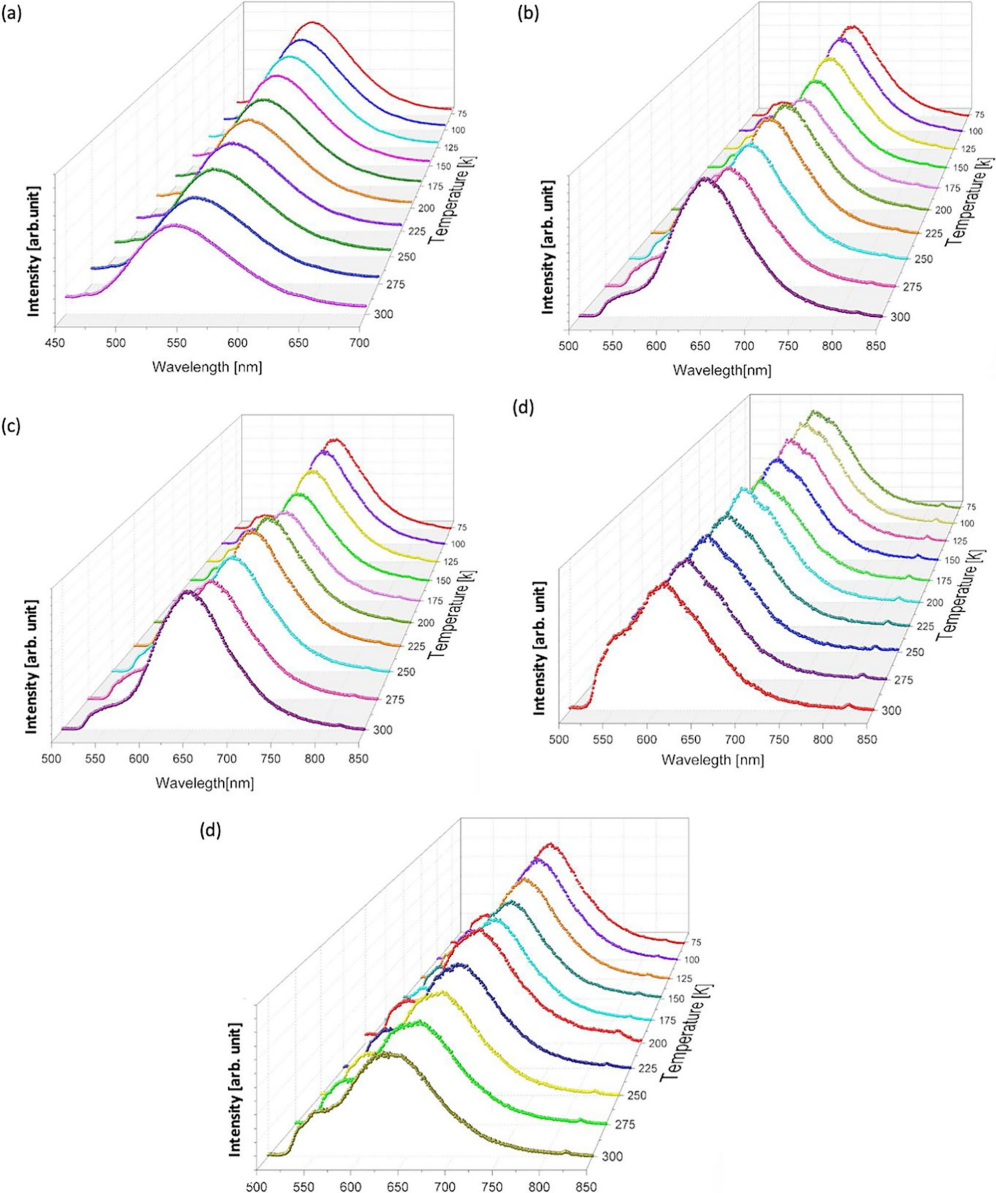
Photoluminescence emission spectra at different temperatures for **a** Znq_2_ pure, **b** DCM pure, **c** 90%DCM10%Znq_2_, **d** 10%DCM90%Znq_2_, **e** 50%DCM50%Znq_2_

The photoluminescence at low temperatures of thin films grown on a glass substrate with different compositions was also studied. Figure 16 shows the photoluminescence of all samples at low temperatures.

The 5%DCM-95%Znq_2_, 10%DCM-90%Znq_2_, and 20%DCM-80%Znq_2_ samples showed a decrease in photoluminescence ranging from 77 to 175 k, then they increased sharply 810^3^ CPS, 5 10^3^ CPS, and 4 10^4^ CPS respectively in the temperature range 200 to 275 K to reach the maximum, then it decreases while keeping the same position: 605 nm for 5%DCM-95%Znq_2_, 600 nm for 10%DCM-90%Znq_2_, and 612 nm for 20%DCM-80%Znq_2_. While for the other samples, the most intense peak is obtained with the lowest temperature (77 k) and as the temperature increases the intensity of the peaks decreases; this decrease is accompanied by a change in the position of the peak maximum (1 nm for each 25 K).

The intensity of the photoluminescence of the elaborated samples is much higher at low temperatures; this dependence on the temperature can be explained by the non-radiative recombination of the photoexcited carriers which becomes more effective when we increase the temperature. Table 3 combines the data for the photoluminescence measurement of all films.

**Table 3:** Results of photoluminescence measurements at low temperatures

	DCM pure		Znq_2_ Pure		5%DCM-95%Znq_2_		10%DCM-90%Znq_2_		20%DCM-80%Znq_2_		50%DCM-50%Znq_2_		70%DCM-30%Znq_2_		90%DCM-10%Znq_2_	
	PL (nm)	EW (CPS)	PL (nm)	EW (CPS)	PL (nm)	EW (CPS)	PL (nm)	EW (CPS)	PL (nm)	EW (CPS)	PL (nm)	EW (CPS)	PL (nm)	EW (CPS)	PL (nm)	EW (CPS)
77	661	2.2 10^5^	537	9.5 10^5^	605	8 10^4^	613	6.5 10^4^	602	8.5 10^4^	648	1 10^5^	659	2.7 10^5^	662	1.5 10^5^
100	660	2.2 10^5^	537	9 10^5^	604	8.6 10^4^	613	6.5 10^4^	602	8.5 10^4^	647	1 10^5^	659	2.7 10^5^	662	1.5 10^5^
125	659	2.1 10^5^	537	8.8 10^5^	604	8.9 10^4^	613	6.4 10^4^	602	8.2 10^4^	645	9.9 10^4^	659	2.6 10^5^	661	1.4 10^5^
150	658	1.9 10^5^	537	8.4 10^5^	604	8.9 10^4^	613	6.2 10^4^	602	8 10^4^	644	9 10^4^	657	2.6 10^5^	658	1.3 10^5^
175	657	1.8 10^5^	539	7.7 10^5^	604	9.5 10^4^	613	6.4 10^4^	602	7.7 10^4^	643	8.9 10^4^	655	2.5 10^5^	656	1.2 10^5^
200	656	1.8 10^5^	540	7.6 10^5^	604	1 10^5^	613	6.6 10^4^	602	1.1 10^5^	641	9.8 10^4^	652	2.7 10^5^	660	1.4 10^5^
225	655	1.7 10^5^	540	7.3 10^5^	604	1 10^5^	613	6.6 10^4^	602	1.2 10^5^	640	8.6 10^4^	652	2.6 10^5^	654	1.5 10^5^
250	652	1.5 10^5^	540	7 10^5^	604	1 10^5^	613	6.6 10^4^	602	1.3 10^5^	639	8.2 10^4^	650	2.6 10^5^	653	1.5 10^5^
275	649	1.4 10^5^	540	6.6 10^5^	604	1 10^5^	613	6.2 10^4^	602	1.3 10^5^	638	7.8 10^4^	649	2.4 10^5^	651	1.5 10^5^
300	648	1.4 10^5^	540	6.4 10^5^	604	1 10^5^	613	6.2 10^4^	595	8.9 10^4^	630	7.5 10^4^	644	2.1 10^5^	644	1.6 10^5^

The most intense photoluminescence is obtained by the pure Znq_2_ sample at low temperature, while for all other samples the DCM character dominates.

### Decay time

Time-resolved photoluminescence (TRPL) is a technique adapted to study the quick electronic deactivation processes that lead to the emission of photons in many types of materials such as metal–organic complexes and dyes. This fluorescence lifetime can be influenced by several parameters such as the molecular environment as well as by interactions with other molecules. This technique makes it possible to measure life in real time.

The lifetime decay of the samples was measured at room temperature (300 K) using the FluoroMax-4 spectrofluorometer, which is connected to the FluoroHub single-photon counting controller using a pulsed diode as the excitation source (Zawadzka et al. 2014). The choice of the diode is related to the excitation wavelength.

**Fig. 17:**
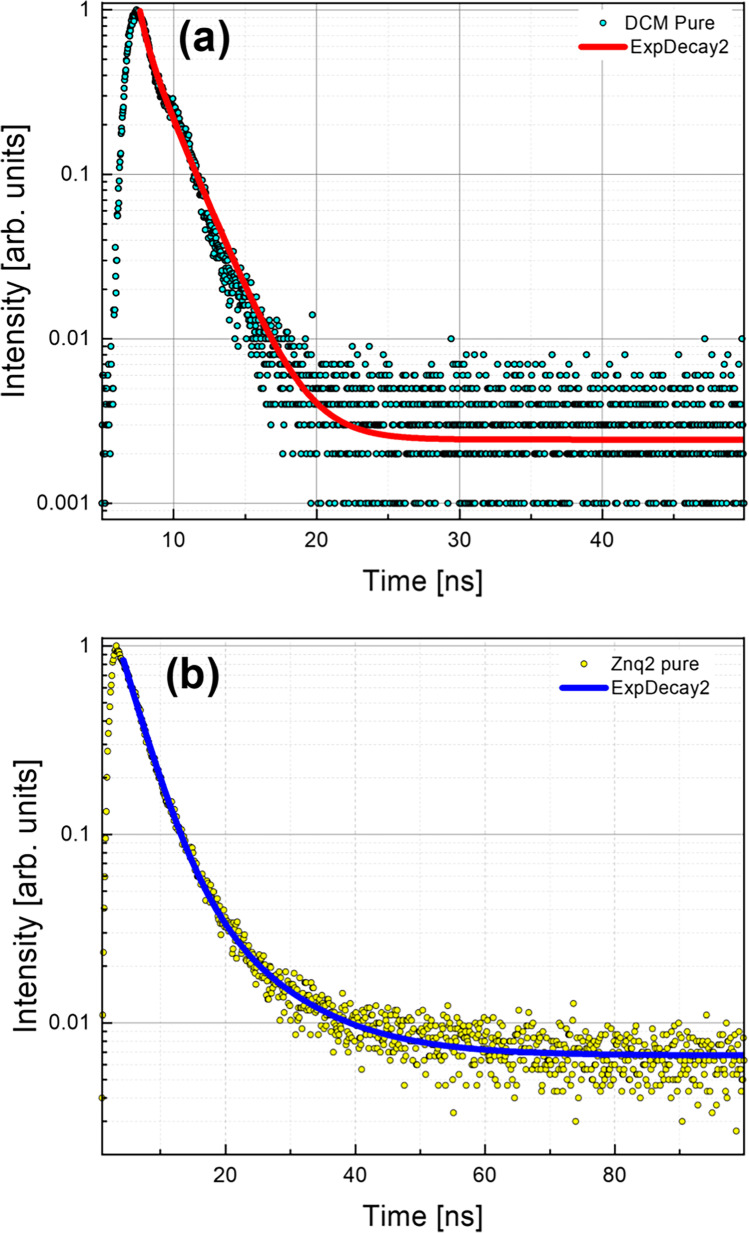
Decay time curves **a** DCM pure and **b** Znq_2_ pure at the maximum of luminescence wavelength at room temperatures of the measurements

**Fig. 18:**
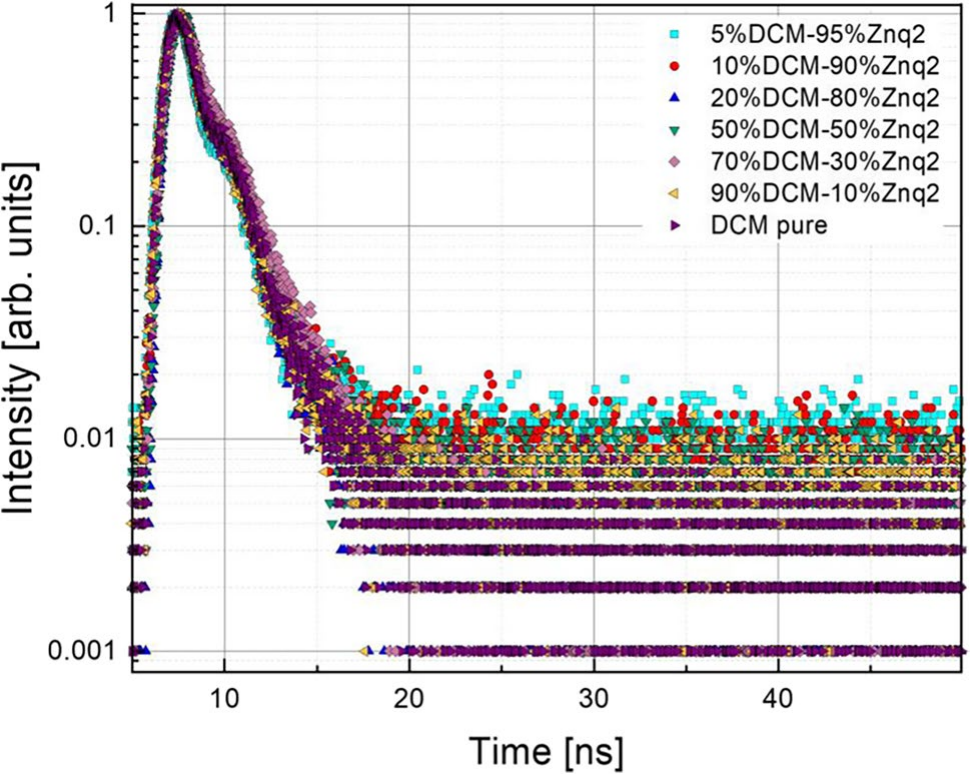
Decay time curves for all the samples at the maximum of luminescence wavelength at room temperatures of the measurements

Figures 17 and 18 show the decay time curves of all studied samples at different compositions. These curves show a transition between the ground state and the excited state and therefore are the results of the interaction between the emitting residues and their excited state of the films.

The spectra of all the films studied showed a decay with a nature of two exponentials. To properly analyze the lifetime decay of the samples, all the curves obtained are fitted with the bi-exponential function described by the following equation:

1 $$Y={A}_{1}{e}^{\frac{t}{{\tau }_{1}}}+{A}_{2}{e}^{\frac{t}{{\tau }_{2}}}$$

where ${A}_{1}$ and ${A}_{2}$ are constants, *t* is pulse time, and ${\tau }_{1}$ and ${\tau }_{2}$ are decay values.

All samples showed two decay time components one short and one long. The Znq_2_ film showed a longer exciton lifetime than the other samples ${\tau }_{1 Znq2}=$ 3.47 ns and ${\tau }_{2 Znq2}=$ 10.96 ns, while the samples with different compositions showed a dominance of the pure DCM film character even in the samples with a high percentage of Znq_2_ as is presented in Fig. 18. The obtained values of the slowest decay components for the other samples are ${\tau }_{1}=0.09$ ns, while the values obtained of the highest decay component are ${\tau }_{2}=2.27$ ns. The average sample lifetime was also calculated ${\tau }_{av}=$ 1.18 ns; consequently, the emission is fluorescence.

In polar environments, the DCM undergoes an intramolecular charge transfer (Kumar Kanaparthi et al. 2021; Lee et al. 2018) but in the following study we work on thin films, and due to the rigid environment hindering the movement of the molecules it is impossible that this phenomenon will occur, and therefore it is very likely that there is a Forster resonance energy transfer phenomena which results in non-radiative energy transfer from the donor fluorophore in the excited state to an acceptor molecule via a Coulomb interaction (Sasaki et al. 2016); this hypothesis can be reinforced by the spectral overlap of the emission spectra of the donor and the absorption spectra of the acceptor. The presence of the two decay times in pure DCM can be explained by two different physical processes illustrated in the transitions that occur from the ground state to the excited state of the donor and the transition from the ground state to the excited state of the acceptor, while for pure Znq_2_ samples, a study suggests that the presence of the two decomposition rates is made up of two distinct physical processes that take place during the transfer of energy from the ground state to a higher state or a central metal atom to a quinoline ligand (Painuly et al. 2020).

## Conclusion

In summary, DCM and Znq_2_, which are red and green luminescent materials, respectively, were deposited under a high vacuum as thin films on glass substrates by the vacuum evaporation method. The emission spectra data shows that a small percentage of DCM inside Znq_2_ can increase the photoluminescence intensity of the film; it also showed that a shift in the peak positions of the samples was also carried out which confirm that a complete energy transfer occurred from Znq_2_ to DCM due to the overlap between the PL emission of Znq_2_ and the absorption of DCM. Excitation spectra data showed in its turns the presence of more than one transition in the different samples. Low-temperature photoluminescence spectra showed that the photoluminescence intensity of the processed samples is much higher at low temperature; this temperature dependence can be explained by the non-radiative recombination of the photoexcited carriers which becomes more efficient when the temperature is increased. The time-resolved photoluminescence (TRPL) technique showed the presence of a single decay time in pure Znq_2_ and two decay times for the other samples. The results obtained show that the combinations of these two materials are a good choice for optoelectronic applications.

## Data Availability

All data generated or analyzed during this study are included in this published article.
